# Supplementary material for: High-Quality Preparation of Energy-Containing Microspheres with Cross-Scale Particle Size
Source: Micromachines (Basel). 2025 Mar 31;16(4):416. doi: 10.3390/mi16040416 (PMC12029273; doi:10.3390/mi16040416)
Supplement: Supplementary file 1 [file micromachines-16-00416-s001.zip › micromachines-3512896-supplementary.pdf]

Supporting Information

High-Quality Preparation of Energy-Containing Microspheres with Cross-Scale Particle Size

Jiang Liu [1,2,†], Hairui Bian [3,†], Guoqiang Yu [1], Jiachao Zhang [4], Yaozheng Wang [4], Dang Ding [3], Ning Sang [3] and Fangsheng Huang [5,*]

[1] Shaanxi Institute of Applied Physical Chemistry, Xi'an 710061, China; liujiang@njust.edu.cn (J.L.); m17791841150@163.com (G.Y.)
[2] School of Chemistry and Chemical Engineering, Nanjing University of Science and Technology, Nanjing 210094, China
[3] Technological Innovation Center of Functional Microcapsule, Hefei Zhongke Create-Micro Technology Co., Ltd., Hefei 230026, China; hairui_bian@163.com (H.B.); dd17352922663@163.com (D.D.); sangning_iat@163.com (N.S.)
[4] School of Instrument Science and Opto-Electronics Engineering, Hefei University of Technology, Hefei 230026, China; 18356514497@163.com (J.Z.); hfut_wyz@163.com (Y.W.)
[5] Institute of Advanced Technology, University of Science and Technology of China, Hefei 230088, China
* Correspondence: huangfs@ustc.edu.cn
† These authors contributed equally to this work.

1. Supporting Figures

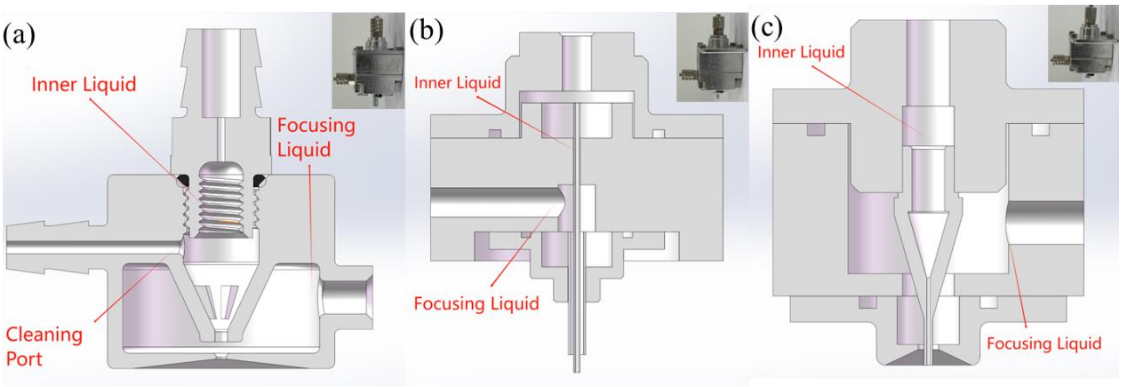

Figure S1. Schematic and physical diagrams of the microfluidic devices: (**a**) Flow-focusing, (**b**) Co-axial flow, (**c**) Pneumatic printing.

The **flow-focusing** device primarily consists of an inner stainless steel needle and a focusing chamber, as illustrated in Figure S1a. The flow-focusing microchannel employs a 24 G/25 G inner needle paired with a focusing chamber featuring a focusing orifice of 500 μm/300 μm in diameter.

The high concentricity between the inner needle and the focusing orifice amplifies the shear forces exerted by the focusing fluid, enabling the inner-phase fluid to form a jet downstream.

A higher-velocity focusing fluid, introduced from the periphery, exerts radial compressive forces on the inner-phase fluid, shaping it into a slender conical jet within the focusing zone. Under the combined action of shear forces and surface tension, the inner

 https://doi.org/10.3390/xxxxx

conical jet undergoes hydrodynamic instability downstream, leading to its breakup into uniform droplets. These droplets are subsequently transported through a receiving conduit, where UV-induced photopolymerization solidifies them into monodisperse microspheres.

The **coaxial flow** device is primarily composed of an inner stainless steel needle and a coaxial focusing chamber, as depicted in Figure S1b.

A 20 G or 22 G inner needle is integrated with a focusing chamber channel of 1700 μm in diameter. The high concentricity between the needle and the chamber ensures that the focusing fluid exerts stable shear forces on the inner-phase fluid, enabling uniform droplet generation. At the outlet of the receiving channel, the droplets undergo UV-induced photopolymerization, ultimately forming monodisperse microspheres.

The **pneumatic printing** device is primarily composed of a stainless steel needle, The microchannel utilizes a 20G/22G inner needle and a focusing chamber channel with a diameter of 1900 μm/1710 μm.

A continuous liquid supply unit, and a pulsed gas supply module, as illustrated in Figure S1c. The inner capillary is supplied with a continuous fluid flow by a precision syringe pump, while the outer capillary delivers pulsed airflow via the controlled opening and closing of a solenoid valve. Under the shear forces induced by the pulsed airflow, droplets are generated at the capillary tip. The solenoid valve enables precise adjustment of the pulsed airflow frequency and pulse width, ensuring consistent droplet formation. Subsequent chemical crosslinking solidification transforms the droplets into monodisperse microspheres.

The microfluidic devices used in this work were fabricated using **well-established soft lithography and CNC machining protocols**, which are widely adopted in microfluidics research. Key steps include:

Mold Design: CAD models of the microchannel networks (e.g., focusing orifices, coaxial nozzles) were generated based on hydrodynamic simulations.

Material Selection: Polydimethylsiloxane (PDMS) was used for soft lithography due to its optical transparency and biocompatibility.

Device Assembly: Channels were bonded to glass substrates via plasma treatment, ensuring leak-free operation.

The microfluidic devices used in this study were fabricated using well-established Computer Numerical Control (CNC) machining protocols, which are widely adopted in microfluidics research. The key steps are as follows:

1. Mold Design: CAD models of the microchannel networks (e.g., focusing orifices, inner-phase and focusing-phase inlets) were generated based on hydrodynamic simulations and extensive practical experience.

2. Material Selection: Components were machined from aerospace-grade aluminum, which offers high precision, durability, and compatibility with microfluidic applications.

3. Device Assembly: The individual components were assembled with high precision to ensure seamless integration and reliable operation.

These steps ensured the robustness and functionality of the microfluidic devices, which are critical for achieving the desired performance in our experiments.
